# Supplementary material for: Potentiation of P2RX7 as a host-directed strategy for control of mycobacterial infection
Source: eLife. 2019 Jan 29;8:e39123. doi: 10.7554/eLife.39123 (PMC6351102; doi:10.7554/eLife.39123)
Supplement: Supplementary file 1. — Compounds with blue background are host directed and compounds in red are known anti-infectives. Asterisk (*) represents compounds with previously identified host-directed anti-mycobacterial activities. [file elife-39123-supp1.pdf]

Drug Name	Approved Use / Target	Other targets	Structure	FMLA structure	Molecular Weight	CAS Number	Therapeutic Class	Therapeutic Effect
Desipramine hydrochloride*	Norepinephrine reuptake inhibitor	Inhibitor of acid sphingomyelinase (1)	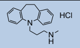	C18H23ClN2	302.85	58-28-6	Central Nervous System	Antidepressant
Chlorpromazine hydrochloride*	Dopamine antagonist	Inhibitor of acid sphingomyelinase (1)	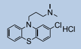	C17H20Cl2N2S	355.33	69-09-0	Cardiovascular	Antiemetic
Trimeprazine tartrate	Antipruritic	increases insulin receptor substrate 2 in islet cells (2)	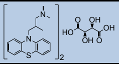	C40H50N4O6S2	747.00	4330-99-8	Allergology	Antihistaminic
Zuclopenthixol dihydrochloride	Dopamine and adrenoceptor antagonist	induces neutropenia (3)	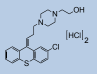	C22H27Cl3N2OS	473.90	633-59-0	Central Nervous System	Antipsychotic
Metixene hydrochloride	Cholinesterase inhibitor	cytotoxic against oral squamous cell carcinoma (4)	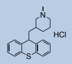	C20H24ClNS	345.94	1553-34-0	Central Nervous System	Antiparkinsonian
Methylhydantoin-5-(L)	Anticonvulsant	inhibits enteroviral replication (5)	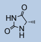	C4H6N2O2	114.10	40856-73-3	Central Nervous System	Anticonvulsant
Equilin	Binds estrogen receptor beta		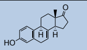	C18H20O2	268.36	474-86-2	Endocrinology	
Clemastine fumarate	Histamine H1 Receptor Antagonist	remyelinates nerves (6) (7), anti-inflammatory (8), multiple sclerosis treatment (9) inhibits HERG K+ channel (10), potentiates P2X7 receptor (11) increases apoptosis (12)	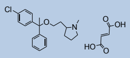	C25H30ClNO5	459.97	14976-57-9	Allergology	Antiemetic
Prothionamide	Thioamide antimycobacterial pro-drug		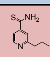	C9H12N2S	180.27	14222-60-7	Infectiology	Antibacterial
Paromomcyin sulfate	aminoglycoside antibiotic		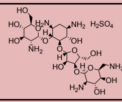	C23H47N5O18S	713.72	1263-89-4	Metabolism	Antiamebic
Spiramycin	macrolide antibiotic		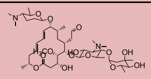	C43H73N2O14R	843.07	8025-81-8	Metabolism	Antibacterial
Enoxacin	fluoroquinolone antibiotic		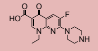	C15H17FN4O3	320.33	84294-96-2	Infectiology	Antibacterial
Sulfamethazine sodium salt	sulfonamide antibacterial		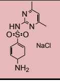	C12H13N4NaO2S	300.32	1981-58-4	Metabolism	Antibacterial

Citations

1) Roca, Francisco J and Lalita Ramakrishnan. “TNF dually mediates resistance and susceptibility to mycobacteria via mitochondrial reactive oxygen species” Cell vol. 153,3 (2013): 521-34.

2) Kuznetsova, Alexandra et al. “Trimeprazine increases IRS2 in human islets and promotes pancreatic β cell growth and function in mice” *JCI insight* vol. 1,3 (2016): e80749.

3) Hirshberg, Boaz, et al. “Zuclopenthixol-Associated Neutropenia and Thrombocytopenia.” Annals of Pharmacotherapy, vol. 34, no. 6, June 2000, pp. 740–742, doi:10.1345/aph.18468.

4) Kellen Cristine Tjioe, Denise Tostes Oliveira & Julie Gavard "Luteolin Impacts on the DNA Damage Pathway in Oral Squamous Cell Carcinoma", Nutrition and Cancer vol. 68,5, (2016): 838-847, DOI: 10.1080/01635581.2016.1180411

5) Yvan Verlinden, Andrea Cuconati, Eckard Wimmer, Bart Rombaut, "The antiviral compound 5-(3,4-dichlorophenyl) methylhydantoin inhibits the post-synthetic cleavages and the assembly of poliovirus in a cell-free system" Antiviral Research Vol 48,1(2000) 61-69

6) Liu, Jia et al. “Clemastine Enhances Myelination in the Prefrontal Cortex and Rescues Behavioral Changes in Socially Isolated Mice” Journal of neuroscience : the official journal of the Society for Neuroscience vol. 36,3 (2016): 957-62.

7) Bruce A C Cree, Jianqin Niu, Kimberly K Hoi, Chao Zhao, Scott D Caganap, Roland G Henry, Dang Q Dao, Daniel R Zollinger, Feng Mei, Yun-An A Shen, Robin J M Franklin, Erik M Ullian, Lan Xiao, Jonah R Chan, Stephen P J Fancy; "Clemastine rescues myelination defects and promotes functional recovery in hypoxic brain injury", Brain, Vol. 141, 1, (2018) 85–98

8) Johansen, Pål et al. "Clemastine causes immune suppression through inhibition of extracellular signal-regulated kinase–dependent proinflammatory cytokines" Journal of Allergy and Clinical Immunology (2011) Vol.128 , 6 , 1286 - 1294

9) Green, Ari J et al. "Clemastine fumarate as a remyelinating therapy for multiple sclerosis (ReBUILD): a randomised, controlled, double-blind, crossover trial"
The Lancet Vol. 390 , 10111 (2017) 2481 - 2489

10) Ridley, John M. et al. "Clemastine, a conventional antihistamine, is a high potency inhibitor of the HERG K+ channel" Journal of Molecular and Cellular Cardiology , Vol. 40,1 (2006) 107 - 118

11) Nörenberg, Wolfgang et al. “Clemastine potentiates the human P2X7 receptor by sensitizing it to lower ATP concentrations” Journal of biological chemistry vol. 286,13 (2011): 11067-81.

12) Döbbeling, U. , Waeckerle-Men, Y. , Zabel, F. , Graf, N. , Kündig, T. M. and Johansen, P. "The antihistamines clemastine and desloratadine inhibit STAT3 and c-Myc activities and induce apoptosis in cutaneous T-cell lymphoma cell lines". Exp Dermatol, 22: (2013) 119-124

Supplementary Table 1: Summary of identified compounds. Compounds with blue background are host directed and compounds in red are known anti-infectives. Asterisk (*) represents compounds with previously identified host-directed anti-mycobacterial activities.
