# Supplementary material for: Potentiation of P2RX7 as a host-directed strategy for control of mycobacterial infection
Source: eLife. 2019 Jan 29;8:e39123. doi: 10.7554/eLife.39123 (PMC6351102; doi:10.7554/eLife.39123)
Supplement: Supplementary file 2. — Statistics performed on transformed data are in yellow and untransformed is in gray. The table is organized by figure number, in chronological order. Statistical tests are listed and transformation equation provided, where applicable. When a paired t-test is performed on paired data, the results of the unpaired t-test are also given. When both paired and unpaired are potentially appropriate (in the case of paired data with ineffective pairing), both test results are given. Statistical test results in italics denote tests not presented within the figures. [file elife-39123-supp2.pdf]

Supplementary Table 2

UNTRANSFORMED DATA							
Figure 2A							
Unpaired t test with Welch's correction							
P value	*0.0016*						
P value summary	**						
Significantly different (P < 0.05)?	*Yes*						
One- or two-tailed P value?	*Two-tailed*						
Welch-corrected t, df	*t=3.343 df=49.38*						
Figure 2C							
Ordinary One-Way ANOVA							
Tukey's multiple comparisons test	*Mean Diff.*	*95.00% CI of diff.*	*Significant?*	*Summary*	*Adjusted P Value*		
0.5% DMSO vs. 1uM CLEM	*13015140*	*-514848 to 26545129*	*No*	*ns*	*0.0637*		*A-B*
0.5% DMSO vs. 5uM CLEM	*16217914*	*2103630 to 30332198*	*Yes*	*	*0.0179*		*A-C*
0.5% DMSO vs. 10uM CLEM	*21799685*	*7448965 to 36150405*	*Yes*	***	*0.0009*		*A-D*
1uM CLEM vs. 5uM CLEM	*3202774*	*-10911510 to 17317057*	*No*	*ns*	*0.9325*		*B-C*
1uM CLEM vs. 10uM CLEM	*8784545*	*-5566176 to 23135265*	*No*	*ns*	*0.3789*		*B-D*
5uM CLEM vs. 10uM CLEM	*5581771*	*-9321101 to 20484642*	*No*	*ns*	*0.7578*		*C-D*
Figure 2E							
Kruskal-Wallis test							
Dunn's multiple comparisons test	*Mean rank diff.*	*Significant?*	*Summary*	*Adjusted P Value*			
DMSO wt/het vs. 5uM CLEM wt/het	*24.4*	*Yes*	*	*0.0352*	*A-B*		
DMSO MUT irf8 vs. 5uM CLEM MUT irf8	*-2.272*	*No*	*ns*	*>0.9999*	*C-D*		
**Figure 2G**							
Unpaired t test							
P value	0.0006						
P value summary	***						
Significantly different (P < 0.05)?	Yes						
One- or two-tailed P value?	Two-tailed						
t, df	t=3.97 df=22						
Figure 3C							
Kruskal-Wallis test							
Dunn's multiple comparisons test	*Mean rank diff.*	*Significant?*	*Summary*	*Adjusted P Value*			
WT DMSO vs. WT Clem	*-35.05*	*Yes*	****	*<0.0001*	*A-B*		
WT DMSO vs. P2RX7^xt26 DMSO	*-8.75*	*No*	*ns*	*>0.9999*	*A-C*		
WT DMSO vs. P2RX7^xt26 DMSO	*-14.4*	*No*	*ns*	*0.7007*	*A-D*		
WT Clem vs. P2RX7^xt26 DMSO	*26.3*	*Yes*	***	*0.0004*	*B-C*		
WT Clem vs. P2RX7^xt26 DMSO	*20.65*	*Yes*	*	*0.0461*	*B-D*		
P2RX7^xt26 DMSO vs. P2RX7^xt26 DMSO	*-5.654*	*No*	*ns*	*>0.9999*	*C-D*		
Figure 4A							
Kruskal-Wallis test							
Dunn's multiple comparisons test	*Mean rank diff.*	*Significant?*	*Summary*	*Adjusted P Value*			
WT DMSO vs. WT CLEM	*36.46*	*Yes*	****	*<0.0001*	*A-B*		
WT DMSO vs. p2x7 mutant DMSO	*16*	*No*	*ns*	*0.7862*	*A-C*		
WT DMSO vs. p2x7 mutant CLEM	*21.38*	*No*	*ns*	*0.4201*	*A-D*		
WT CLEM vs. p2x7 mutant DMSO	*-20.46*	*No*	*ns*	*0.3208*	*B-C*		
WT CLEM vs. p2x7 mutant CLEM	*-15.08*	*No*	*ns*	*>0.9999*	*B-D*		
p2x7 mutant DMSO vs. p2x7 mutant CLEM	*5.383*	*No*	*ns*	*>0.9999*	*C-D*		
Figure 5A							
Kruskal-Wallis test							
Dunn's multiple comparisons test	*Mean rank diff.*	*Significant?*	*Summary*	*Adjusted P Value*			
Mm:WT DMSO vs. Mm:WT CLEM	*29.12*	*Yes*	*	*0.043*	*A-B*		
Mm:WT DMSO vs. Mm:ΔRD1 DMSO	*36.31*	*Yes*	**	*0.0044*	*A-C*		
Mm:WT DMSO vs. Mm:ΔRD1 CLEM	*36.49*	*Yes*	**	*0.0014*	*A-D*		
Mm:WT CLEM vs. Mm:ΔRD1 DMSO	*7.195*	*No*	*ns*	*>0.9999*	*B-C*		
Mm:WT CLEM vs. Mm:ΔRD1 CLEM	*7.373*	*No*	*ns*	*>0.9999*	*B-D*		
Mm:ΔRD1 DMSO vs. Mm:ΔRD1 CLEM	*0.1776*	*No*	*ns*	*>0.9999*	*C-D*		
Figure 5B							
Kruskal-Wallis test							
Dunn's multiple comparisons test	*Mean rank diff.*	*Significant?*	*Summary*	*Adjusted P Value*			
DMSO WT/HET vs. CLEM WT/HET	*21.52*	*Yes*	*	*0.0285*	*A-B*		
DMSO WT/HET vs. DMSO ASC MUTANT	*-16.56*	*No*	*ns*	*0.3995*	*A-C*		
DMSO WT/HET vs. CLEM ASC MUTANT	*-12.91*	*No*	*ns*	*>0.9999*	*A-D*		
CLEM WT/HET vs. DMSO ASC MUTANT	*-38.08*	*Yes*	***	*0.0001*	*B-C*		
CLEM WT/HET vs. CLEM ASC MUTANT	*-34.43*	*Yes*	**	*0.0073*	*B-D*		
DMSO ASC MUTANT vs. CLEM ASC MUTANT	*3.649*	*No*	*ns*	*>0.9999*	*C-D*		
**Figure 6B**							

TRANSFORMED DATA						
**Figure 2A**	y=log(y)					
Unpaired t test						
P value	0.0001					
P value summary	***					
Significantly different (P < 0.05)?	Yes					
One- or two-tailed P value?	Two-tailed					
t, df	t=3.997 df=84					
**Figure 2C**	y=log(y)					
Ordinary One-Way ANOVA						
Tukey's multiple comparisons test	Mean Diff.	95.00% CI of diff.	Significant?	Summary	Adjusted P Value	
0.5% DMSO vs. 1uM CLEM	0.2452	-0.02369 to 0.514	No	ns	0.0864	A-B
0.5% DMSO vs. 5uM CLEM	0.346	0.0655 to 0.6264	Yes	**	0.0095	A-C
0.5% DMSO vs. 10uM CLEM	0.5622	0.2718 to 0.8526	Yes	****	<0.0001	A-D
1uM CLEM vs. 5uM CLEM	0.1008	-0.1797 to 0.3813	No	ns	0.7799	B-C
1uM CLEM vs. 10uM CLEM	0.317	0.02665 to 0.6075	Yes	*	0.027	B-D
5uM CLEM vs. 10uM CLEM	0.2162	-0.08494 to 0.5174	No	ns	0.2415	C-D
**Figure 2E**	y=log(y)					
Kruskal-Wallis test						
Dunn's multiple comparisons test	Mean rank diff.	Significant?	Summary	Adjusted P Value		
DMSO wt/het vs. 5uM CLEM wt/het	24.4	Yes	*	0.0352	A-B	
DMSO MUT irf8 vs. 5uM CLEM MUT irf8	-2.272	No	ns	>0.9999	C-D	
**Figure 2G**						
N/A						
**Figure 3C**	y=y/30					
Kruskal-Wallis test						
Dunn's multiple comparisons test	Mean rank diff.	Significant?	Summary	Adjusted P Value		
WT DMSO vs. WT Clem	-35.05	Yes	****	<0.0001	A-B	
WT DMSO vs. P2RX7^xt26 DMSO	-8.75	No	ns	>0.9999	A-C	
WT DMSO vs. P2RX7^xt26 DMSO	-14.4	No	ns	0.7007	A-D	
WT Clem vs. P2RX7^xt26 DMSO	26.3	Yes	***	0.0004	B-C	
WT Clem vs. P2RX7^xt26 DMSO	20.65	Yes	*	0.0461	B-D	
P2RX7^xt26 DMSO vs. P2RX7^xt26 DMSO	-5.654	No	ns	>0.9999	C-D	
**Figure 4A**	y=log(y)					
Ordinary One-Way ANOVA						
Tukey's multiple comparisons test	Mean Diff.	95.00% CI of diff.	Significant?	Summary	Adjusted P Value	
WT DMSO vs. WT CLEM	0.6204	0.3006 to 0.9402	Yes	****	<0.0001	A-B
WT DMSO vs. p2x7 mutant DMSO	0.3088	-0.1204 to 0.7379	No	ns	0.2451	A-C
WT DMSO vs. p2x7 mutant CLEM	0.382	-0.09595 to 0.86	No	ns	0.1652	A-D
WT CLEM vs. p2x7 mutant DMSO	-0.3117	-0.7408 to 0.1174	No	ns	0.2375	B-C
WT CLEM vs. p2x7 mutant CLEM	-0.2384	-0.7164 to 0.2396	No	ns	0.5662	B-D
p2x7 mutant DMSO vs. p2x7 mutant CLEM	0.07327	-0.4838 to 0.6303	No	ns	0.9862	C-D
**Figure 5A**	y=log(y)					
Ordinary One-Way ANOVA						
Tukey's multiple comparisons test	Mean Diff.	95.00% CI of diff.	Significant?	Summary	Adjusted P Value	
Mm:WT DMSO vs. Mm:WT CLEM	0.3101	0.02344 to 0.5967	Yes	*	0.0284	A-B
Mm:WT DMSO vs. Mm:ΔRD1 DMSO	0.3712	0.08659 to 0.6558	Yes	**	0.0049	A-C
Mm:WT DMSO vs. Mm:ΔRD1 CLEM	0.3655	0.1034 to 0.6276	Yes	**	0.0022	A-D
Mm:WT CLEM vs. Mm:ΔRD1 DMSO	0.06113	-0.2213 to 0.3436	No	ns	0.943	B-C
Mm:WT CLEM vs. Mm:ΔRD1 CLEM	0.05543	-0.2043 to 0.3151	No	ns	0.9452	B-D
Mm:ΔRD1 DMSO vs. Mm:ΔRD1 CLEM	-0.005705	-0.2632 to 0.2518	No	ns	>0.9999	C-D
**Figure 5B**	y=log(y)					
Ordinary One-Way ANOVA						
Tukey's multiple comparisons test	Mean Diff.	95.00% CI of diff.	Significant?	Summary	Adjusted P Value	
DMSO WT/HET vs. CLEM WT/HET	0.241	0.03425 to 0.4477	Yes	*	0.0154	A-B
DMSO WT/HET vs. DMSO ASC MUTANT	-0.152	-0.3969 to 0.09285	No	ns	0.3723	A-C
DMSO WT/HET vs. CLEM ASC MUTANT	-0.1423	-0.4329 to 0.1483	No	ns	0.5798	A-D
CLEM WT/HET vs. DMSO ASC MUTANT	-0.393	-0.6358 to -0.1503	Yes	***	0.0003	B-C
CLEM WT/HET vs. CLEM ASC MUTANT	-0.3833	-0.6721 to -0.09447	Yes	**	0.0042	B-D
DMSO ASC MUTANT vs. CLEM ASC MUTANT	0.009765	-0.3075 to 0.327	No	ns	0.9998	C-D
**Figure 6B**						

Supplementary Table 2

"Friedman test" ANOVA					
Dunn's multiple comparisons test	Rank sum diff.	Significant?	Summary	Adjusted P Value	
WT DMSO vs. WT CLEM	16	Yes	**	0.0021	A-B
WT DMSO vs. P2X7 mutant DMSO	6	No	ns	>0.9999	A-C
WT DMSO vs. P2X7 mutant CLEM	10	No	ns	0.1521	A-D
WT CLEM vs. P2X7 mutant DMSO	-10	No	ns	0.1521	B-C
WT CLEM vs. P2X7 mutant CLEM	-6	No	ns	>0.9999	B-D
P2X7 mutant DMSO vs. P2X7 mutant CLEM	4	No	ns	>0.9999	C-D

N/A

**Figure 6C**					
Paired t test				*Unpaired t test with Welch's correction*	
P value	0.0253			*P value*	*0.0253*
P value summary	*			*P value summary*	*
Significantly different (P < 0.05)?	Yes			*Significantly different (P < 0.05)?*	*Yes*
One- or two-tailed P value?	Two-tailed			*One- or two-tailed P value?*	*Two-tailed*
t, df	t=4.157 df=3			*Welch-corrected t, df*	*t=4.157 df=3*
Number of pairs	4				

**Figure 6C**
N/A

Figure 6D					
Paired t test	*pairing values: r=0.6966, p=0.0028*			*Unpaired t test with Welch's correction*	
P value	*<0.0001*			*P value*	*<0.0001*
P value summary	****			*P value summary*	****
Significantly different (P < 0.05)?	*Yes*			*Significantly different (P < 0.05)?*	*Yes*
One- or two-tailed P value?	*Two-tailed*			*One- or two-tailed P value?*	*Two-tailed*
t, df	*t=7.625 df=13*			*Welch-corrected t, df*	*t=6.227 df=15.36*
Number of pairs	*14*				

**Figure 6D**	y=log(y)				
Paired t test	pairing values: r=0.6966, p=0.0028			*Unpaired t test with Welch's correction*	
P value	<0.0001			*P value*	*<0.0001*
P value summary	****			*P value summary*	****
Significantly different (P < 0.05)?	Yes			*Significantly different (P < 0.05)?*	*Yes*
One- or two-tailed P value?	Two-tailed			*One- or two-tailed P value?*	*Two-tailed*
t, df	t=9.965 df=13			*Welch-corrected t, df*	*t=6.389 df=20.23*
Number of pairs	14				

**Figure 6E**	*note: one outlier was excluded. Only 2 colonies grew on the least diluted plate despite having high bacterial values by fluorescence. It was >2 SD Log(0.8) away from the mean Log(5.23)
Unpaired t test with Welch's correction	
P value	0.2392
P value summary	ns
Significantly different (P < 0.05)?	No
One- or two-tailed P value?	Two-tailed
Welch-corrected t, df	t=1.381 df=4.01

**Figure 6E**	y=log(y)	*note: one outlier was excluded. Only 2 colonies grew on the least diluted plate despite having high bacterial values by fluorescence. It was >2 SD Log(0.8) away from the mean Log(5.23)
Unpaired t test		
P value	0.0037	
P value summary	**	
Significantly different (P < 0.05)?	Yes	
One- or two-tailed P value?	Two-tailed	
t, df	t=3.877 df=9	

Figure 6E without outlier removed	
Unpaired t test with Welch's correction	
P value	*0.2366*
P value summary	*ns*
Significantly different (P < 0.05)?	*No*
One- or two-tailed P value?	*Two-tailed*
Welch-corrected t, df	*t=1.344 df=5.018*

Figure 6E without outlier removed	
Unpaired t test	
P value	*0.1397*
P value summary	*ns*
Significantly different (P < 0.05)?	*No*
One- or two-tailed P value?	*Two-tailed*
t, df	*t=1.605 df=10*

Figure 6F					
Kruskal-Wallis test					
Dunn's multiple comparisons test	*Mean rank diff.*	*Significant?*	*Summary*	*Adjusted P Value*	
AB DMSO (0.5%) vs. AB CLEM (5uM)	*21.76*	*Yes*	*	*0.011*	*A-B*
AB DMSO (0.5%) vs. AB MOX (2ug/mL)	*28.56*	*Yes*	***	*0.0003*	*A-C*
AB DMSO (0.5%) vs. AB MOX (2ug/mL)+ CLEM (5uM)	*49.67*	*Yes*	****	*<0.0001*	*A-D*
AB CLEM (5uM) vs. AB MOX (2ug/mL)	*6.801*	*No*	*ns*	*>0.9999*	*B-C*
AB CLEM (5uM) vs. AB MOX (2ug/mL)+ CLEM (5uM)	*27.92*	*Yes*	***	*0.0002*	*B-D*
AB MOX (2ug/mL) vs. AB MOX (2ug/mL)+ CLEM (5uM)	*21.11*	*Yes*	*	*0.0131*	*C-D*

**Figure 6F**	y=log(y)				
Kruskal-Wallis test					
Dunn's multiple comparisons test	Mean rank diff.	Significant?	Summary	Adjusted P Value	
AB DMSO (0.5%) vs. AB CLEM (5uM)	21.76	Yes	*	0.011	A-B
AB DMSO (0.5%) vs. AB MOX (2ug/mL)	28.56	Yes	***	0.0003	A-C
AB DMSO (0.5%) vs. AB MOX (2ug/mL)+ CLEM (5uM)	49.67	Yes	****	<0.0001	A-D
AB CLEM (5uM) vs. AB MOX (2ug/mL)	6.801	No	ns	>0.9999	B-C
AB CLEM (5uM) vs. AB MOX (2ug/mL)+ CLEM (5uM)	27.92	Yes	***	0.0002	B-D
AB MOX (2ug/mL) vs. AB MOX (2ug/mL)+ CLEM (5uM)	21.11	Yes	*	0.0131	C-D

Supplementary Table 2: Summary of p values and statistical tests for Figures 1-6. Statistics performed on transformed data are in yellow and untransformed is in gray. The table is organized by figure number, in chronological order. Statistical tests are listed and transformation equation provided, where applicable. When a paired t-test is performed on paired data, the results of the unpaired t-test are also given. When both paired and unpaired are potentially appropriate (in the case of paired data with ineffective pairing), both test results are given. Statistical test results in *italics* denote tests not presented within the figures.
