# Supplementary material for: Potentiation of P2RX7 as a host-directed strategy for control of mycobacterial infection
Source: eLife. 2019 Jan 29;8:e39123. doi: 10.7554/eLife.39123 (PMC6351102; doi:10.7554/eLife.39123)
Supplement: Supplementary file 3. — Statistics performed on transformed data are in orange and untransformed is in light blue. The table is organized by figure number, in chronological order. Statistical tests are listed and transformation equation provided, where applicable. When a paired t-test is performed on paired data, the results of the unpaired t-test are also given. When both paired and unpaired are potentially appropriate (in the case of paired data with ineffective pairing), both test results are given. Statistical test results in italics denote tests not presented within the figure. [file elife-39123-supp3.pdf]

Supplementary Table 3: Summary of p values and statistical tests for Supplementary Figures 1-9. Statistics performed on transformed data are in orange and untransformed is in light blue. The table is organized by figure number, in chronological order. Statistical tests are listed and transformation equation provided, where applicable. When a paired t-test is performed on paired data, the results of the unpaired t-test are also given. When both paired and unpaired are potentially appropriate (in the case of paired data with ineffective pairing), both test results are given. Statistical test results in *italics* denote tests not presented within the figure.

Supplementary Table 3

UNTRANSFORMED							TRANSFORMED						
**Supplementary Figure 2A**							**Supplementary Figure 2A**	y=log(y)					
Unpaired t test with Welch's correction							Unpaired t test						
P value	0.1304						P value	0.3147					
P value summary	ns						P value summary	ns					
Significantly different (P < 0.05)?	No						Significantly different (P < 0.05)?	No					
One- or two-tailed P value?	Two-tailed						One- or two-tailed P value?	Two-tailed					
Welch-corrected t, df	t=1.528 df=81.05						t, df	t=1.011 df=96					

**Supplementary Figure A**							**Supplementary Figure 2A**						
Unpaired t test							N/A						
P value	0.1907												
P value summary	ns												
Significantly different (P < 0.05)?	No												
One- or two-tailed P value?	Two-tailed												
t, df	t=1.35 df=22												

**Supplementary Figure 2E**							**Supplementary Figure 2E**						
Kruskal-Wallis test													
Dunn's multiple comparisons test	Mean rank diff.	Significant?	Summary	Adjusted P Value									
1dpi dmso vs. 1dpi clem	7.895	No	ns	>0.9999	A-B								
2dpi dmso vs. 2dpi clem	3.268	No	ns	>0.9999	C-D								
3dpi dmso vs. 3dpi clem	3.926	No	ns	>0.9999	E-F								
4dpi dmso vs. 4dpi clem	-17.82	No	ns	>0.9999	G-H								
5dpi dmso vs. 5dpi clem	28.72	No	ns	0.5505	I-J								

**Supplementary Figure 3A**							**Supplementary Figure 3A**	y=y/30					
Ordinary One-Way ANOVA							Ordinary One-Way ANOVA						
Tukey's multiple comparisons test	Mean Diff.	95.00% CI of diff.	Significant?	Summary	Adjusted P Value		Tukey's multiple comparisons test	Mean Diff.	95.00% CI of diff.	Significant?	Summary	Adjusted P Value	
WT1 vs. WT2	-0.1125	-1.255 to 1.03	No	ns	0.9997	A-B	WT1 vs. WT2	-0.00375	-0.04184 to 0.03434	No	ns	0.9997	A-B
WT1 vs. WT3	-0.2125	-1.494 to 1.069	No	ns	0.9964	A-C	WT1 vs. WT3	-0.007083	-0.04981 to 0.03564	No	ns	0.9964	A-C
WT1 vs. WT4	0.02083	-1.501 to 1.543	No	ns	>0.9999	A-D	WT1 vs. WT4	0.0006944	-0.05005 to 0.05144	No	ns	>0.9999	A-D
WT1 vs. WT5	-0.5625	-1.777 to 0.6518	No	ns	0.7472	A-E	WT1 vs. WT5	-0.01875	-0.05923 to 0.02173	No	ns	0.7472	A-E
WT1 vs. WT6	0.0875	-1.542 to 1.717	No	ns	>0.9999	A-F	WT1 vs. WT6	0.002917	-0.05139 to 0.05722	No	ns	>0.9999	A-F
WT2 vs. WT3	-0.1	-1.398 to 1.198	No	ns	>0.9999	B-C	WT2 vs. WT3	-0.003333	-0.04661 to 0.03994	No	ns	>0.9999	B-C
WT2 vs. WT4	0.1333	-1.403 to 1.669	No	ns	0.9998	B-D	WT2 vs. WT4	0.004444	-0.04676 to 0.05564	No	ns	0.9998	B-D
WT2 vs. WT5	-0.45	-1.682 to 0.7815	No	ns	0.8886	B-E	WT2 vs. WT5	-0.015	-0.05605 to 0.02605	No	ns	0.8886	B-E
WT2 vs. WT6	0.2	-1.442 to 1.842	No	ns	0.9992	B-F	WT2 vs. WT6	0.006667	-0.04807 to 0.0614	No	ns	0.9992	B-F
WT3 vs. WT4	0.2333	-1.409 to 1.875	No	ns	0.9983	C-D	WT3 vs. WT4	0.007778	-0.04696 to 0.06251	No	ns	0.9983	C-D
WT3 vs. WT5	-0.35	-1.712 to 1.012	No	ns	0.9734	C-E	WT3 vs. WT5	-0.01167	-0.05705 to 0.03372	No	ns	0.9734	C-E
WT3 vs. WT6	0.3	-1.442 to 2.042	No	ns	0.9957	C-F	WT3 vs. WT6	0.01	-0.04806 to 0.06806	No	ns	0.9957	C-F
WT4 vs. WT5	-0.5833	-2.173 to 1.007	No	ns	0.8868	D-E	WT4 vs. WT5	-0.01944	-0.07244 to 0.03355	No	ns	0.8868	D-E
WT4 vs. WT6	0.06667	-1.859 to 1.992	No	ns	>0.9999	D-F	WT4 vs. WT6	0.002222	-0.06196 to 0.0664	No	ns	>0.9999	D-F
WT5 vs. WT6	0.65	-1.043 to 2.343	No	ns	0.8661	E-F	WT5 vs. WT6	0.02167	-0.03475 to 0.07809	No	ns	0.8661	E-F

**Supplementary Figure 3B**							**Supplementary Figure 3B**	y=y/30					
Unpaired t test with Welch's correction							Unpaired t test with Welch's correction						
P value	0.3206						P value	0.3206					
P value summary	ns						P value summary	ns					
Significantly different (P < 0.05)?	No						Significantly different (P < 0.05)?	No					
One- or two-tailed P value?	Two-tailed						One- or two-tailed P value?	Two-tailed					
Welch-corrected t, df	t=1.008 df=33.6						Welch-corrected t, df	t=1.008 df=33.6					

**Supplementary Figure 3C**							**Supplementary Figure 3C**	y=y/30					
Kruskal-Wallis test							Kruskal-Wallis test						
Dunn's multiple comparisons test	Mean rank diff.	Significant?	Summary	Adjusted P Value			Dunn's multiple comparisons test	Mean rank diff.	Significant?	Summary	Adjusted P Value		
WT DMSO vs. WT CLEM	-9.55	No	ns	0.2407	A-B		WT DMSO vs. WT CLEM	-9.55	No	ns	0.2407	A-B	
WT DMSO vs. P2RX7^xt26 DMSO	1.036	No	ns	>0.9999	A-C		WT DMSO vs. P2RX7^xt26 DMSO	1.036	No	ns	>0.9999	A-C	
WT DMSO vs. P2RX7^xt26 CLEM	-2.375	No	ns	>0.9999	A-D		WT DMSO vs. P2RX7^xt26 CLEM	-2.375	No	ns	>0.9999	A-D	
WT CLEM vs. P2RX7^xt26 DMSO	10.59	No	ns	0.3357	B-C		WT CLEM vs. P2RX7^xt26 DMSO	10.59	No	ns	0.3357	B-C	
WT CLEM vs. P2RX7^xt26 CLEM	7.175	No	ns	>0.9999	B-D		WT CLEM vs. P2RX7^xt26 CLEM	7.175	No	ns	>0.9999	B-D	
P2RX7^xt26 DMSO vs. P2RX7^xt26 CLEM	-3.411	No	ns	>0.9999	C-D		P2RX7^xt26 DMSO vs. P2RX7^xt26 CLEM	-3.411	No	ns	>0.9999	C-D	

**Supplementary Figure 4A**							**Supplementary Figure 4A**						
Repeated measures ANOVA summary							N/A						
Tukey's multiple comparisons test	Mean Diff.	95.00% CI of diff.	Significant?	Summary	Adjusted P Value								

Supplementary Table 3

DMSO WT vs. CLEM WT	0.5875	0.4064 to 0.7686	Yes	****	<0.0001	A-B							
DMSO WT vs. DMSO MUT	0.3203	0.04111 to 0.5995	Yes	*	0.0238	A-C							
DMSO WT vs. CLEM MUT	0.4017	0.07144 to 0.732	Yes	*	0.0168	A-D							
CLEM WT vs. DMSO MUT	-0.2672	-0.5597 to 0.02539	No	ns	0.0769	B-C							
CLEM WT vs. CLEM MUT	-0.1857	-0.4811 to 0.1096	No	ns	0.2855	B-D							
DMSO MUT vs. CLEM MUT	0.08142	-0.1797 to 0.3425	No	ns	0.7854	C-D							

**Supplementary Figure 4B**							**Supplementary Figure 4B**						
Paired t test	pairing values: r=0.7341, p=0.0033			*Unpaired t test*			N/A						
P value	0.0013			*P value*	*0.0157*								
P value summary	**			*P value summary*	*								
Significantly different (P < 0.05)?	Yes			*Significantly different (P < 0.05)?*	*Yes*								
One- or two-tailed P value?	Two-tailed			*One- or two-tailed P value?*	*Two-tailed*								
t, df	t=4.279 df=11			*t, df*	*t=2.618 df=22*								
Number of pairs	12												

Supplementary Figure 5B							**Supplementary Figure 5B**	y=log(y)					
Kruskal-Wallis test							Ordinary One-Way ANOVA						
Dunn's multiple comparisons test	*Mean rank diff.*	*Significant?*	*Summary*	*Adjusted P Value*			Sidak's multiple comparisons test	Mean Diff.	95.00% CI of diff.	Significant?	Summary	Adjusted P Value	
DMSO WT vs. CLEM WT	*44.11*	*Yes*	*	*0.0336*	*A-B*		DMSO WT vs. CLEM WT	0.3804	0.02332 to 0.7375	Yes	*	0.0327	A-B
cmaA2 DMSO vs. cmaA2 clem	*30.4*	*No*	*ns*	*0.0835*	*C-D*		cmaA2 DMSO vs. cmaA2 clem	0.4527	0.1689 to 0.7366	Yes	***	0.0005	C-D
RD1 DMSO vs. RD1 CLEM	*12.18*	*No*	*ns*	*>0.9999*	*E-F*		RD1 DMSO vs. RD1 CLEM	0.09292	-0.2386 to 0.4245	No	ns	0.8757	E-F

**Supplementary Figure 5C**							**Supplementary Figure 5C**						
Unpaired t test	unpaired because there are only two pairs and cannot test for effective pairing						N/A						
P value	0.04												
P value summary	*												
Significantly different (P < 0.05)?	Yes												
One- or two-tailed P value?	Two-tailed												
t, df	t=4.851 df=2												

**Supplementary Figure 5D**							**Supplementary Figure 5D**						
Ordinary One-Way ANOVA							N/A						
Holm-Sidak's multiple comparisons test	Mean Diff.	Significant?	Summary	Adjusted P Value									
wt dmso vs. WT clem	0.1442	Yes	*	0.0468	A-B								
wt dmso vs. RD1 DMSO	0.2869	Yes	****	<0.0001	A-C								
wt dmso vs. RD1 clem	0.2431	Yes	***	0.0002	A-D								
WT clem vs. RD1 DMSO	0.1428	Yes	*	0.0468	B-C								
WT clem vs. RD1 clem	0.09895	No	ns	0.1495	B-D								
RD1 DMSO vs. RD1 clem	-0.04381	No	ns	0.4243	C-D								

**Supplementary Figure 6C**				*Paired t-test*	*pairing values: r=0.1413, p=0.4549*		**Supplementary Figure 6C**						
Unpaired t test	pairing was ineffective, so unpaired was performed			*P value*	*0.0606*		N/A						
P value	0.0197			*P value summary*	*ns*								
P value summary	*			*Significantly different (P < 0.05)?*	*No*								
Significantly different (P < 0.05)?	Yes			*One- or two-tailed P value?*	*Two-tailed*								
One- or two-tailed P value?	Two-tailed			*t, df*	*t=3.875 df=2*								
t, df	t=3.764 df=4			*Number of pairs*	*3*								
**Supplementary Figure 6D**							**Supplementary Figure 6D**						
Unpaired t test							N/A						
P value	0.4585												
P value summary	ns												
Significantly different (P < 0.05)?	No												
One- or two-tailed P value?	Two-tailed												
t, df	t=0.7596 df=16												

Supplementary Table 3

Supplementary Figure 7A							**Supplementary Figure 7A**						
Kruskal-Wallis test							Ordinary One-Way ANOVA	y=log(y)					
Dunn's multiple comparisons test	*Mean rank diff.*	*Significant?*	*Summary*	*Adjusted P Value*			Tukey's multiple comparisons test	Mean Diff.	95.00% CI of diff.	Significant?	Summary	Adjusted P Value	
DMSO wt vs. CLEM wt	*25.96*	*Yes*	*	*0.0216*	*A-B*		DMSO wt vs. CLEM wt	1.543	0.01631 to 3.07	Yes	*	0.0467	A-B
DMSO wt vs. DMSO del	*11.71*	*No*	*ns*	*0.1957*	*A-C*		DMSO wt vs. DMSO del	0.7455	-0.1928 to 1.684	No	ns	0.1635	A-C
DMSO wt vs. CLEM del	*18.4*	*Yes*	**	*0.0084*	*A-D*		DMSO wt vs. CLEM del	0.7654	-0.2201 to 1.751	No	ns	0.179	A-D
CLEM wt vs. DMSO del	*-14.25*	*No*	*ns*	*0.5891*	*B-C*		CLEM wt vs. DMSO del	-0.7974	-2.272 to 0.6775	No	ns	0.4829	B-C
CLEM wt vs. CLEM del	*-7.563*	*No*	*ns*	*>0.9999*	*B-D*		CLEM wt vs. CLEM del	-0.7776	-2.283 to 0.7277	No	ns	0.522	B-D
DMSO del vs. CLEM del	*6.688*	*No*	*ns*	*>0.9999*	*C-D*		DMSO del vs. CLEM del	0.01983	-0.8833 to 0.923	No	ns	>0.9999	C-D

**Supplementary Figure 7B**							**Supplementary Figure 7B**						
Paired t test	pairing values: r=0.7491, p=0.043			*Unpaired t test with Welch's correction*	*Unequal variances*		N/A						
P value	0.0339			*P value*	*0.0738*								
P value summary	*			*P value summary*	*ns*								
Significantly different (P < 0.05)?	Yes			*Significantly different (P < 0.05)?*	*No*								
One- or two-tailed P value?	Two-tailed			*One- or two-tailed P value?*	*Two-tailed*								
t, df	t=2.897 df=5			*Welch-corrected t, df*	*t=2.157 df=6.076*								
Number of pairs	6												

**Supplementary Figure 8B**							**Supplementary Figure 8B**						
Paired t test	pairing values: r=0.8872, p=0.056			*Unpaired t test*			N/A						
P value	0.0075			*P value*	*0.0564*								
P value summary	**			*P value summary*	*ns*								
Significantly different (P < 0.05)?	Yes			*Significantly different (P < 0.05)?*	*No*								
One- or two-tailed P value?	Two-tailed			*One- or two-tailed P value?*	*Two-tailed*								
t, df	t=6.456 df=3			*t, df*	*t=2.157 df=10*								
Number of pairs	4												

Supplementary Figure 8C							**Supplementary Figure 8C**	y=log(y)					
Unpaired t test	*pairing was ineffective, un-paired t-test was performed*			*Paired t test*	*pairing values: r=0.6598, p=0.1701*		Unpaired t test	Pairing was ineffective so unpaired t-test was performed		*Paired t test*	*pairing values: r=0.6835, p=-.1583*		
P value	*0.8566*			*P value*	*0.7853*		P value	0.8875		*P value*	*0.8262*		
P value summary	*ns*			*P value summary*	*ns*		P value summary	ns		*P value summary*	*ns*		
Significantly different (P < 0.05)?	*No*			*Significantly different (P < 0.05)?*	*No*		Significantly different (P < 0.05)?	No		*Significantly different (P < 0.05)?*	*No*		
One- or two-tailed P value?	*Two-tailed*			*One- or two-tailed P value?*	*Two-tailed*		One- or two-tailed P value?	Two-tailed		*One- or two-tailed P value?*	*Two-tailed*		
t, df	*t=0.1886 df=6*			*t, df*	*t=0.2978 df=3*		t, df	t=0.1476 df=6		*t, df*	*t=0.2394 df=3*		
				Number of pairs	*4*					*Number of pairs*	*4*		

**Supplementary Figure 9C**							**Supplementary Figure 9C**						
Repeated measures ANOVA summary							N/A						
Tukey's multiple comparisons test	Mean Diff.	95.00% CI of diff.	Significant?	Summary	Adjusted P Value								
CLEM vs. MOX	-0.05336	-0.3689 to 0.2622	No	ns	0.647	A-B							
CLEM vs. MOX+Clem	0.0724	-0.1554 to 0.3002	No	ns	0.342	A-C							
MOX vs. MOX+Clem	0.1258	0.01737 to 0.2341	Yes	*	0.0376	B-C							
