# Supplementary material for: Potentiation of P2RX7 as a host-directed strategy for control of mycobacterial infection
Source: eLife. 2019 Jan 29;8:e39123. doi: 10.7554/eLife.39123 (PMC6351102; doi:10.7554/eLife.39123)
Supplement: Transparent reporting form [file elife-39123-transrepform.docx]

***eLife’s* transparent reporting form**

We encourage authors to provide detailed information *within their submission* to facilitate the interpretation and replication of experiments. Authors can upload supporting documentation to indicate the use of appropriate reporting guidelines for health-related research (see [EQUATOR Network](http://www.equator-network.org/%20)), life science research (see the [BioSharing Information Resource](https://biosharing.org/)), or the [ARRIVE guidelines](http://www.plosbiology.org/article/info:doi/10.1371/journal.pbio.1000412) for reporting work involving animal research. Where applicable, authors should refer to any relevant reporting standards documents in this form.

If you have any questions, please consult our Journal Policies and/or contact us: [editorial@elifesciences.org](mailto:editorial@elifesciences.org).

**Sample-size estimation**

- You should state whether an appropriate sample size was computed when the study was being designed
- You should state the statistical method of sample size computation and any required assumptions
- If no explicit power analysis was used, you should describe how you decided what sample (replicate) size (number) to use

Please outline where this information can be found within the submission (e.g., sections or figure legends), or explain why this information doesn’t apply to your submission:

In the figure legends we provide information about the number of animals used and the number of times each experiment was repeated, as well as the statistical methods used to determine significance within each figure panel.

Based on previous work using zebrafish to study mycobacterial infection, we have determined that between 10 and 30 animals per treatment group leads to reproducible, statistically powerful analyses.

“group” is defined by a treatment and genotype (carrier-treated wildtype or drug-treated mutant)

For most figures, the number of animals used in each experiment can be directly counted from the points on the graph. These figures are:

Figure 2A, 2C,2E,2G, 3C,4A, 5A, 5B

Figure 2-figure supplement 1A,C,E, Figure 5-figure supplement 1B,D, Figure 5-figure supplement 2C.

For experiments using granuloma explants, each dot typically represents a single granuloma and the sample size can be directly counted in Figure 6F and Figure 6-figure supplement 1A.

In figures involving calcium signaling, fewer animals are used because the effect being measured is within cells and time-intensive microscopy limits the number of animals that can be imaged. The number of animals is at least 3 per group, with at least 4 cells counted from each animal. If a cell remains in view during the whole video and flashes at least once, it is counted.

Figure 3B: 3-5 animals per group.

Figure 3-figure supplement 1A: 6 animals with at least 5 cells counted per animal

Figure 3-figure supplement 1B: At least 3 animals per group

Figure 3-figure supplement 1C: Same number of animals counted as Figure 3B. These are the cells that are uninfected from those same animals.

Other figures show the averages of experiments. The sample sizes for these experiments are detailed below:

Figure 6B (and Figure 6-figure supplement 1B): 6 experiments with at least 4 granulomas per group in each experiment.

Figure 6C, Figure 6-figure supplement 2 B,C : 4 experiments with at least 3 technical replicates of 10 granulomas per replicate.

Figure 6D, Figure 6-figure supplement 3A (left y-axis): 13 experiments with at least 4 granulomas per group in each experiment.

Figure 6E, Figure 6-figure supplement 3A (right y-axis): at least 5 granulomas were plated individually per group.

Figure 4-figure supplement 1A and B: 12 experiments with at least 15 animal per group in each experiment.

Figure 4-figure supplement 1C: 25 animals were pooled for 16s RNA qPCR.

Figure 5-figure supplement 1C: Left y-axis represents 2 experiments with at least 20 animals per group in each experiment. Right axis represents 25 pooled animals for 16S rRNA qPCR.

Figure 6-figure supplement 3C: three independent experiments with at least 8 granulomas per group in each experiment.

**Replicates**

- You should report how often each experiment was performed
- You should include a definition of biological versus technical replication
- The data obtained should be provided and sufficient information should be provided to indicate the number of independent biological and/or technical replicates
- If you encountered any outliers, you should describe how these were handled
- Criteria for exclusion/inclusion of data should be clearly stated
- High-throughput sequence data should be uploaded before submission, with a private link for reviewers provided (these are available from both GEO and ArrayExpress)

Please outline where this information can be found within the submission (e.g., sections or figure legends), or explain why this information doesn’t apply to your submission:

In each figure legend, we provide the number of times the experiment was performed. Each independent experiment represents a biological replicate, meaning that the animals are born on different days (from different parents) and infected with a different aliquot of bacteria unless otherwise noted.

Figure 1B: representative of three biological replicates (experiments) with at least 15 animals per group in each experiment.

Figure 2A: representative of at least 5 biological replicates (independent experiments) with at least 20 animals per group in each experiment. Each dot represents an animal.

Figure 2C: representative of three biological replicates (independent experiments) with at least 20 animals per group in each experiment. Each dot represents an animal.

Figure 2D: Bacterial growth curves are the average of 3 technical replicates (plate readings) and two biological replicates (independent experiments).

Figure 2E: Pooled results of two experiments of three biological replicates (independent experiments) with at least 10 animals per group in each experiment. Each dot represents an animal.

Figure 2G and Figure 2-figure supplement 1C: Represents three biological replicates (independent experiments) with at least 10 animals per group in each experiment. Each dot represents an animal.

Figure 3C and Figure3-figure supplement 1: All results from at least three biological replicates per group. Each biological replicate is a single animal. Individual cell behavior within each animal is being monitored, each dot represents a cell.

Figure 4A: representative of 12 biological replicates (experiments) with at least 15 animals per group in each experiment. Each dot represents an animal.

Figure 5A: representative of three biological replicates (experiments) with at least 15 animals per group in each experiment. Each dot represents an animal.

Figure 5B: representative of three biological replicates (experiments) with at least 12 animals per group in each experiment. Each dot represents an animal.

Figure 6B: Shows the averages of 6 biological replicates (independent experiments) with at least 4 granulomas per group in each experiment. Each dot represents an experiment.

Figure 6C: Shows averages of 4 biological replicates (experiments) with at least 3 technical replicates of 10 granulomas per replicate. Each biological replicate contains granulomas from a different animal. Each dot represents an experiment.

Figure 6D, Figure 6-figure supplement 3A: Shows averages of 13 biological replicates (experiments) with at least 4 granulomas per group in each experiment. Each dot represents an experiment.

Figure 6E: Shows the pooled results of two experiments with three biological replicates (granulomas culture *ex vivo* in individual wells). Each dot represents a granuloma.

Figure 6F: Pooled results of two experiments of three biological replicates (independent experiments) with at least 8 granulomas per group in each experiment. Each dot represents a granuloma.

Continued:

Figure 1-figure supplement 1A-D: Bacterial growth curves are the average of 3 technical replicates (plate readings) and two biological replicates (independent experiments).

Figure 2-figure supplement 1A: representative of two biological replicates (experiments) with at least 25 animals per group in each experiment. Each dot represents an animal.

Figure 2-figure supplement 1E: representative of two biological replicates (experiments) with at least 15 animals per group in each experiment. The same animals were counted each day, but due to different microscope settings between days, only data within each day can be compared. Each dot represents an animal.

Figure 3-figure supplement 1A: Shows the results of 6 biological replicates (animals). Each dot represents a cell.

Figure 3-figure supplement 1B: All results from at least three biological replicates per group. Each biological replicate is a single animal, imaged at different times, on different days, with a different bacterial inoculum. Individual cell behavior within each animal is being monitored, each dot represents a cell.

Figure 4-figure supplement 1A, B: Summary of 12 biological replicates (experiments) with at least 15 animals per group in each experiment. Each dot represents an experiment.

Figure 4-figure supplement 1C: 25 animals were used in each group to create one biological replicate of qPCR, performed in technical triplicate.

Figure 5-figure supplement 1B: representative of two biological replicates (experiments) with at least 15 animals per group in each experiment. Each dot represents an animal.

Figure 5-figure supplement 1C: (left y-axis) summary of 2 biological replicates (experiments) with at least 15 animals per group. Each dot represents an experiment. Right y-axis is from 25 animals in each group to create one biological replicate of qPCR, performed in technical triplicate.

Figure 5-figure supplement 1D: Representative of 3 experiments with at least 10 animals per group in each experiment. Each dot represents an animal.

Figure 5-figure supplement 2C: Representative of two biological replicates (experiments) with at least 5 animals per group in each experiment. Because these experiments require time-intensive microscopy, fewer animals can be analyzed within the doubling time of mycobacteria. Each dot represents an animal.

Figure 6-figure supplement 1A: Representative of 6 experiments (shown in Figure 6B and Figure 6-figure supplement 1B). Each dot represents a granuloma.

Figure 6-figure supplement 1B: Summary of 6 experiments. Each dot represents an experiment.

Continued:

Figure 6-figure supplement 2B,C: Summary of four experiments (biological replicates). Each experiment contained 3-4 technical replicates, which were composed of 5-10 granulomas from the same animal, exposed to the same treatment regimen and kept in the same wells. Each dot represents an experiment.

Figure 6-figure supplement 3B: Bacterial growth curves are the average of 3 technical replicates (plate readings) from the same tube (1 biological replicate) within each group.

Figure 6-figure supplement 3C: Summary of three experiments with at least 8 granulomas per experiment. Each dot represents an experiment.

**Statistical reporting**

- Statistical analysis methods should be described and justified
- Raw data should be presented in figures whenever informative to do so (typically when N per group is less than 10)
- For each experiment, you should identify the statistical tests used, exact values of N, definitions of center, methods of multiple test correction, and dispersion and precision measures (e.g., mean, median, SD, SEM, confidence intervals; and, for the major substantive results, a measure of effect size (e.g., Pearson's r, Cohen's d)
- Report exact p-values wherever possible alongside the summary statistics and 95% confidence intervals. These should be reported for all key questions and not only when the p-value is less than 0.05.

Please outline where this information can be found within the submission (e.g., sections or figure legends), or explain why this information doesn’t apply to your submission:

All statistical test summary information can be found in **Supplementary Table 2** (main figures) and **Supplementary Table 3** (Supplementary figures). These include all tests performed on data presented as well as untransformed data, if transformed data was presented within the figure. Within the figure, all statistically significant p-values are presented. Within the figure legend, both the non-statistically significant p-values and the statistical test (with proper correction, if applicable) are provided. Statistical analyses are also described briefly in the materials and methods section.

(For large datasets, or papers with a very large number of statistical tests, you may upload a single table file with tests, Ns, etc., with reference to sections in the manuscript.)

**Group allocation**

- Indicate how samples were allocated into experimental groups (in the case of clinical studies, please specify allocation to treatment method); if randomization was used, please also state if restricted randomization was applied
- Indicate if masking was used during group allocation, data collection and/or data analysis

Please outline where this information can be found within the submission (e.g., sections or figure legends), or explain why this information doesn’t apply to your submission:

Animals were allocated into treatment groups randomly. When animals are infected, each genotype or bacterial strain-based group was pooled and then randomly sorted into ‘treatment’ or ‘control’, blind (masked) to initial infection burden. When possible, heterozygous incrosses of animals were performed and thus all imaging and quantification were performed blind (masked) to genotype (Figures 2E, 4A, 5B) with genotyping performed post-hoc.

In all calcium-related experiments (Figure 3 and Figure 3-figure supplement 1), counts for calcium flashes were performed blind (masked) to genotype and treatment.

All granulomas were pooled by animal genotype and randomly sorted into ‘treatment’ and ‘control’ wells. The bacterial burdens within granulomas is highly variable and therefore we remained blind (masked) to infection burden until after treatment groups had been assigned.

**Additional data files (“source data”)**

- We encourage you to upload relevant additional data files, such as numerical data that are represented as a graph in a figure, or as a summary table
- Where provided, these should be in the most useful format, and they can be uploaded as “Source data” files linked to a main figure or table
- Include model definition files including the full list of parameters used
- Include code used for data analysis (e.g., R, MatLab)
- Avoid stating that data files are “available upon request”

Please indicate the figures or tables for which source data files have been provided:

We have supplied the results of all statistical tests on all data in Supplementary Tables 2 and 3 (including parallel tests on transformed and untransformed data).

As described above, we present raw data in most figures where each dot represents the exact numerical value for the bacterial burden within one animal.
